# Innovative methods for involving people with dementia and carers in the policymaking process

**DOI:** 10.1111/hex.13213

**Published:** 2021-03-02

**Authors:** Fiona Keogh, Patricia Carney, Eamon O’Shea

**Affiliations:** ^1^ Centre for Economic and Social Research on Dementia National University of Ireland Galway Galway Ireland; ^2^ Department of Public Health Midlands, Health Service Executive Tullamore Ireland

**Keywords:** carers, co‐production, dementia, film, illustrations, policy

## Abstract

**Background:**

There are few examples of public patient involvement in policymaking for groups whose ability to participate may be affected by a disability, particularly for people with dementia and their family carers. Principles of engagement and inclusion in democratic processes are as important for these groups as other citizens. We used two innovative methods to increase involvement of people with dementia and family carers in the policymaking process in Ireland, specifically in relation to impending legislation on home care.

**Method:**

A Policy Café was co‐produced with people with dementia using a World Café methodology. A Carer's Assembly was co‐produced with caregivers of people with dementia using a citizen's assembly model.

**Results:**

Ten people with dementia discussed policy developments they wanted to see implemented in relation to diagnosis and home care. Twenty‐eight dementia caregivers prioritized four topics: home care; respite services; psychosocial supports; and financial supports. Film and illustrations were used to create accessible messages for policy‐makers to inform their decision making.

**Discussion:**

Involving people with dementia and carers in policy development requires time and creativity to facilitate and maximize their involvement. Co‐production is essential to ensure the priorities of participants are identified, expressed and communicated effectively. A cumulative sequence of events and messages can generate significant engagement with policy‐makers. Policy‐makers need to hear the direct and authentic voice of people with dementia and carers when faced with important policy decisions.

**Patient or public contribution:**

People with dementia and carers were involved in study design, data analysis and presentation.

## 1 INTRODUCTION

In the past two decades, much has been achieved in moving public and patient involvement (PPI) in health research from being a marginal or niche activity to one which is a necessary criterion for receiving research funding.^1^, ^2^ The involvement of patients and citizens in health research and policy is supported by legal and regulatory requirements^3^ as well as having a moral and pragmatic footing.^4^ Progress has been made in the involvement of people with dementia in research, particularly in the last decade.^5^ Organizations such as the Dementia Engagement and Empowerment Project,^6^ the Alzheimer Society of Ireland^7^ and others have produced practical guidance on how best to involve people with dementia in research. Self‐advocates such as Wendy Mitchell have been particularly strong on the two‐way benefits of involvement in research and the personal benefits and benefits to the research process and research outcomes.^8^

Public involvement in health system planning and policymaking is increasingly recognized as a core activity in many industrialized countries, with an assumption that greater engagement by citizens in governance leads to more democratic and effective processes.^9^, ^10^, ^11^, ^12^, ^13^ Patient and family engagement, in particular, is seen as offering a pathway towards better‐quality health care, more‐efficient care and improved population health.^14^ The latter outline a framework for patient and family engagement in health care at three levels: direct care; organizational design and governance; and policymaking. However, sometimes there are downsides to citizen participation, not least time and cost, and it does not always produce the desired results, either for the citizen participants or government.^15^

While there is growing evidence of patient engagement in dementia research, there is less evidence of the involvement of people with dementia or family carers in the policymaking process. A scoping review of the literature in preparation for the work reported here found no published journal papers on the involvement of people with dementia in the policymaking process. A report in the grey literature described the engagement of people with dementia in the development of 20 National Dementia Plans and Strategies, describing a variety of types of engagement in the policymaking process from consultation processes to being members of the policymaking group.^16^ Where engagement does occur, it is often through advocacy organizations. It is important not to confuse advocacy and PPI; the latter connects the person with dementia and the policy‐maker directly, while advocacy remains an indirect relationship, even when expertly communicated and delivered.

A variety of mechanisms are used to support citizen engagement in policymaking, from quite simple approaches such as a written submission, to more sophisticated methods such as citizen juries.^11^ However, most methods are not readily accessible to the majority of people with dementia, or indeed to members of other marginalized groups.^17^, ^18^ Nonetheless, policy is where the ‘big decisions’ are made which affect people with dementia every day in terms of the allocation of funding to health and social care services, as well as other areas such as transport, legal rights and more.

Until recently, health policymaking in Ireland has been largely a top‐down process with little direct involvement of the public and patient voice. People with dementia were involved in the development of Ireland's National Dementia Strategy (NDS)^19^ (DoH, 2014) indirectly, through a facilitated consultation process and the inclusion of the national advocacy organization (ASI) on the policymaking group. While progress has been made in the implementation of some actions in the NDS, such as raising awareness and staff training,^20^ there is frustration at the slow implementation of measures which would more directly benefit people with dementia.^21^ Although personhood was referenced as an overarching principle in the NDS, much clearer direction on how personhood and person‐centred care can be supported in practice and through regulation is necessary in Ireland.^22^ More direct and impactful methods of involving people with dementia in policymaking and policy implementation are necessary to ensure their concerns and priorities can be more rapidly realized in service delivery.

As with many other researchers, a central focus of our work has been to involve people with dementia centrally in the work of our research centre and to have maximal impact on dementia policy in Ireland.^23^, ^24^ Our understanding of how best to achieve both aims has developed over the past 5 years, in particular our appreciation that involving people with dementia does not just yield benefits in terms of the relevance of research questions and methods, but also provides a pathway to greater policy impact. Focused national funding to develop PPI in research has aided the involvement of a range of patient groups, including people with dementia.^25^

It has always been a challenge to involve patients whose condition can create additional barriers to involvement, such as people with communication difficulties; people whose capacity is challenged; or people whose cognitive abilities affect their understanding of conceptual or complicated information. However, there are many examples of PPI in research which overcome these barriers.^5^, ^26^, ^27^ The use of creative and innovative methods can be particularly useful for addressing barriers to involvement for people with different disabilities. In this paper, we describe two such methods for involving people with dementia and their carers in the policymaking process which combine the principles of co‐production and PPI; a dementia policy café and a citizen's assembly for carers. The aim of this work was to create a pathway for the voice and experiences of people with dementia and family carers to influence upcoming legislation on home care—currently represented through advocacy but not through direct voice. We could identify no examples in the literature of the use of such approaches with people with dementia and caregivers to influence the policymaking process.

## 2 METHOD

### 2.1 Policy café

Over a number of years, the authors had discussed and explored with people with dementia, pathways through which they and their peers could be more directly involved in the policymaking process in a way which would empower them, present a forum for their views but also have a real impact on policy. The idea of a policy café was one which started to take shape as it was an accessible and engaging format and would allow for the direct capture of the voice and experience of people with dementia. The World Café is a participatory method with a flexible format that can be adapted to different circumstances. Seven design principles are set out by the World Café Community Foundation,^28^ which emphasize generating an informal, hospitable, creative space, encouraging and valuing everyone's contribution and identifying insights. Issues are discussed at round café tables, with a small number of participants for a set period of time and the insights from each table are shared with the larger group. The policy café method used in this study was adapted from the World Café model.

#### 2.1.1 Participants

The Irish Dementia Working Group (IDWG) is an advocacy group of people with a diagnosis of dementia who work to share their experiences and highlight issues important to them. The IDWG formed in 2012 and now has four regional groups with a total of 40 active members. Twelve members of the group were approached by the advocacy organization (ASI) that supports the group, to identify their willingness to be involved in the policy café. Using the knowledge of the ASI facilitator of the group, potential participants were selected based on their availability and their ability to participate in the exercise for the whole event. An information sheet was sent to each person and their carer/supporter. Two of those approached were not available and ten members agreed to participate, five females and five males, from urban and rural environments, including people with young‐onset and later‐onset dementia.

#### 2.1.2 Topic identification and development

**FIGURE 1:**
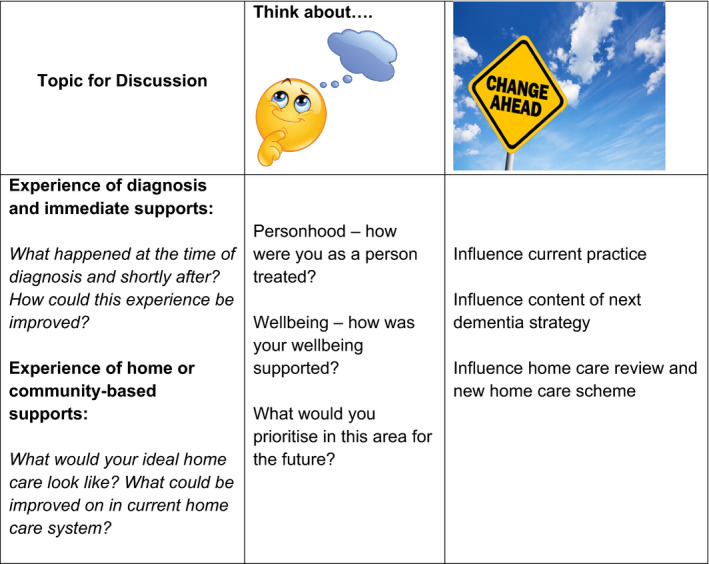
Topics and questions discussed at the Policy Café

The topics to be discussed were identified through examining consultations and materials which members of the IDWG had helped produce for other work. Discussions were held through (pre‐Covid19) face‐to‐face meetings and phone calls with two members of the IDWG to identify topics and associated questions to be addressed in the policy café and two topics were identified: home care and diagnosis with post‐diagnostic supports (PDS). Home care is a current and high priority policy area as the Department of Health in Ireland is currently engaged in a review of the funding and regulation of home care as part of an ongoing legislative programme of reform.^29^ The development of diagnostic facilities and processes in Ireland is a high priority for the National Dementia Office and is also the subject of discussions around budgetary planning at a national level. Therefore, there were identifiable forums (such as planning committees and review groups) with whom to communicate the issues identified from the café and they were timely and high priority issues for policy‐makers and decision‐makers as well as people with dementia. As recommended in the World Café guidelines, questions were developed from these topics and were posted on the wall of the meeting room and on each table (see Figure 1).

#### 2.1.3 The event

A hotel venue was selected which was familiar to the participants, and additional touches were made to create an informal, hospitable space through the use of round tables, tablecloths, flowers and pictures. Materials to draw and record the discussions were on each table. The café commenced with a brief presentation to set the context and how the café would run. There were two tables with five participants each. A facilitator was also at each table not to chair the discussion but to prompt participants as to the questions, to assist in capturing the discussions and to provide support to participants who needed it. The café ran for 2.5 hours with a break halfway. One table discussed home care and the other, diagnosis and post‐diagnostic support. Each topic was discussed generally for a period, and then, each person was prompted to write down their own priorities on cards to be put in a bowl in the centre of the table. Participants then sorted these priorities into common themes and a final set of priority messages was recorded for each table—home care and diagnosis. This process was used to ensure that each person had direct input into generating ideas or priorities and to ensure there was no ‘filtering’ of messages by a note taker and that the words of the participants were directly used in framing each priority. After the discussion of each topic, the participants from each table swapped tables to review the priorities identified by the other table and to add to them. This step of reviewing the insights and outputs of other tables is an integral part of the World Café method. An artist was commissioned to provide real‐time illustration of the discussions, and a film maker was also present to film the proceedings.

### 2.2 Carers’ Assembly

The Citizens’ Assembly in Ireland has been described as a deliberative mini‐public characterized by two main features; first, participants are randomly selected to be representative of the wider population and second, the mode of operation which consists of presentations by experts in the topic area, followed by round table discussions and a ballot on policy options.^30^ The Citizens’ Assembly methodology was adapted to run a similar type of forum for family carers of people with dementia, to discuss issues of concern to them and to identify priorities to bring to policy‐makers about to make important legislative decisions on the future provision of home care in Ireland. The objectives of the Carers Assembly were to formulate a list of priority actions to support family carers of people with dementia and to rank priorities for public policy identified by participants of the Assembly.

#### 2.2.1 Participants

There is no readily available database of dementia caregivers from which we could randomly select participants for a Carer's Assembly. Instead, participants were recruited through three non‐governmental organizations: the Alzheimer Society of Ireland, Family Carers Ireland and Western Alzheimer's. In addition, we drew on a mailing list of carers who had expressed an interest in being involved in research studies compiled by the research centre of the authors. In the recruitment process, attempts were made to include current and former carers of people with dementia, family carers providing care to people at different stages of dementia, and varied relationships to the person to whom they provide care, age and gender.

Thirty‐two carers agreed to attend. Four family carers had to withdraw at the last minute for various reasons and 28 carers took part in the Assembly meeting. Participants were mainly from the West of Ireland with a small number travelling from the East of the country. Of the attendees, 18 per cent were male, 28 per cent currently or previously cared for a spouse or partner, 68 per cent provided care one or more parents and 7 per cent cared for a sibling.

#### 2.2.2 Preparations

Following a series of preliminary meetings and discussions with carer organizations, individual meetings with carers and two focus group meetings with 12 carers, four topics were selected for discussion at the Assembly: home care, respite, social supports and financial support (see Table 1 for topic descriptions). A number of experts were identified to make brief presentations on these topics at the Assembly. The itinerary for the Carers' Assembly was agreed by a steering group made up of current and former family carers of people with dementia, policy and academic experts. The principles for the day were as follows: openness and transparency; fairness and equality of voice; efficiency; and respect, collegiality and confidentiality. An advisory panel consisting of six individuals that work for carer support organizations in Ireland and academics was also brought together for the day to provide clarity on technical issues that might arise, for example in relation to existing supports, regulation and resource allocation funding models. The advisory panel also developed the ballot papers to be voted on by participants following the roundtable discussions. Two ‘pilot runs’ of the Assembly were organized to ensure that logistics would work and that it would be possible to prepare ballot papers in time for voting at the end of the Assembly. Following the pilot, a number of changes were made to the agenda and plan for the day, including the following: change in the schedule for the day to increase the focus on carers and give them the opportunity to engage more; allowing for flexibility with timings on the day; carefully planned spacing of tables so that the atmosphere was collegial while allowing people to hear discussions at their table; and ensuring all language and descriptions provided were as simple and accessible as possible.

**TABLE 1:** Topic descriptions for Carer's Assembly

Topic	Brief description
Home Care	What is meant by a quality homecare service and what are the main elements that make a quality service? Home care was defined as care that is provided in the home by a professionally trained carer
Respite Services	Services which aim to improve the well‐being of carers and people with dementia through the provision of short breaks for carers
Emotional and social well‐being supports	Supports and services for the emotional and social well‐being of family carers of people with dementia
Financial Supports	Supports for the financial and economic well‐being of family carers of people with dementia

#### 2.2.3 The event

The Assembly was a day‐long meeting in a hotel room chosen for its natural light and friendly ambience. Participants were arranged in circular tables of five to six people. At each table, there was a trained facilitator and a note taker. The role of the facilitator was to ensure that roundtable discussions kept to the point and were respectful and that every member had an opportunity to speak.

Academic experts gave a brief presentation on each topic. After the four presentations, there was an opportunity for questions. Each topic was then considered separately in a round table discussion managed by a facilitator. The note taker recorded the main discussion points taking note of the main issues agreed by carers during the discussion that should be included on the ballot paper. These notes were then shared with an expert advisory panel who developed a ballot based on these discussions. Carers then voted on issues raised during the Assembly to rank their preferences for supports under each heading, thereby helping to establish priorities for dementia care in Ireland. In a final ballot, participants were also asked to rank the overall topics addressed on the day in order of importance to them.

### 2.3 Ethics

Ethical approval for the policy café and the carer's assembly was obtained from the Research Ethics Committee in the National University of Ireland, Galway, #18‐Dec‐12. Written consent was obtained from each person for participation in their respective events and for filming. It was possible to participate without providing consent for filming, as plans were in place to frame the filming in ways which excluded participants who did not wish to be filmed. Consent to be filmed was obtained from all participants in the policy café and from 23 participants in the Carer's Assembly. All methods were carried out in accordance with relevant guidelines and regulations.

## 3 RESULTS

### 3.1 Policy café

The messages for each topic were generated by the participants. The priorities for home care focused on the process and quality of home care (see Table 2), as well as the need for other services such as transport and for information and support for family carers. The importance of rights and funding was also emphasized so that the homecare priorities could be implemented.

**TABLE 2:** Priorities for home care identified by policy café participants

Priorities for policy‐makers identified by participants: Home care
Model of home care: social health Help with activities and community involvement Tailored to the person, look beyond symptoms, provides the assistance the person wants and needs Flexible and responsive Support to keep healthy, connected, active and social Support ‘in time and at the right time’ Our voices and our choices Vital role of the community for the continuum of care Pathway of care to be clear and integrated at all stages Carers to be trained/skilled (eg empathetic, confidential, sensitive, understand dementia and comorbidities) Information and support for family carers Adapting the house for the person Transport is essential Ring‐fenced funding—allocate resources to need Strong policy and Bill of Rights for dementia (very important for those under 65 years)

The priorities for diagnosis and PDS focused on the need for personalized diagnosis and proactively linking people to PDS, as well as the importance of stigma reduction to encourage people with dementia to come forward for diagnosis (see Table 3). Rights and citizenship were also important priorities related to this topic.

**TABLE 3:** Priorities for diagnosis and PDS identified by policy café participants

Priorities for policy‐makers identified by participants: Diagnosis and PDS
Public Awareness Reduce stigma (and fear‐based stigma) Increase training Personalized Diagnosis Trained personnel to make and impart diagnosis Ask person and family what their challenges are Give hope, use appropriate language Enable and empower person Proactively link person to services after diagnosis Personalized and appropriate information Age‐appropriate information/services Social Supports (appropriate) peer‐support Normalized living Interdisciplinary team (including person themselves) and dementia diagnosis support team Timely diagnosis Right to know diagnosis Not knowing prevents obtaining services Citizenship Part of society Inclusive Being part of our community Conflict between being dementia focused and being inclusive

Participant's views of the process were sought throughout the morning and revealed elements of the method which worked well and those which did not work so well. Participants enjoyed the creative elements, such as writing down ideas themselves and the illustrations. They also welcomed the informality, the opportunity to discuss the topics and enjoyed the venue and the refreshments. However, moving to the next table to review the ideas generated on the alternative topic did not work so well. Participants wanted to discuss this topic fully themselves rather than just review what had been generated by the other group and add to that. As a consequence, some felt there was not enough time for this part of the discussions. We also had to change the tablecloths as the pattern was visually disruptive for some individuals. Participants' views were also captured in three questions at the end of the discussions to which nine participants responded; eight found the event useful for getting views across; seven thought there was enough time to join the conversations and eight thought it was a useful way of having a conversation.

**FIGURE 2:**
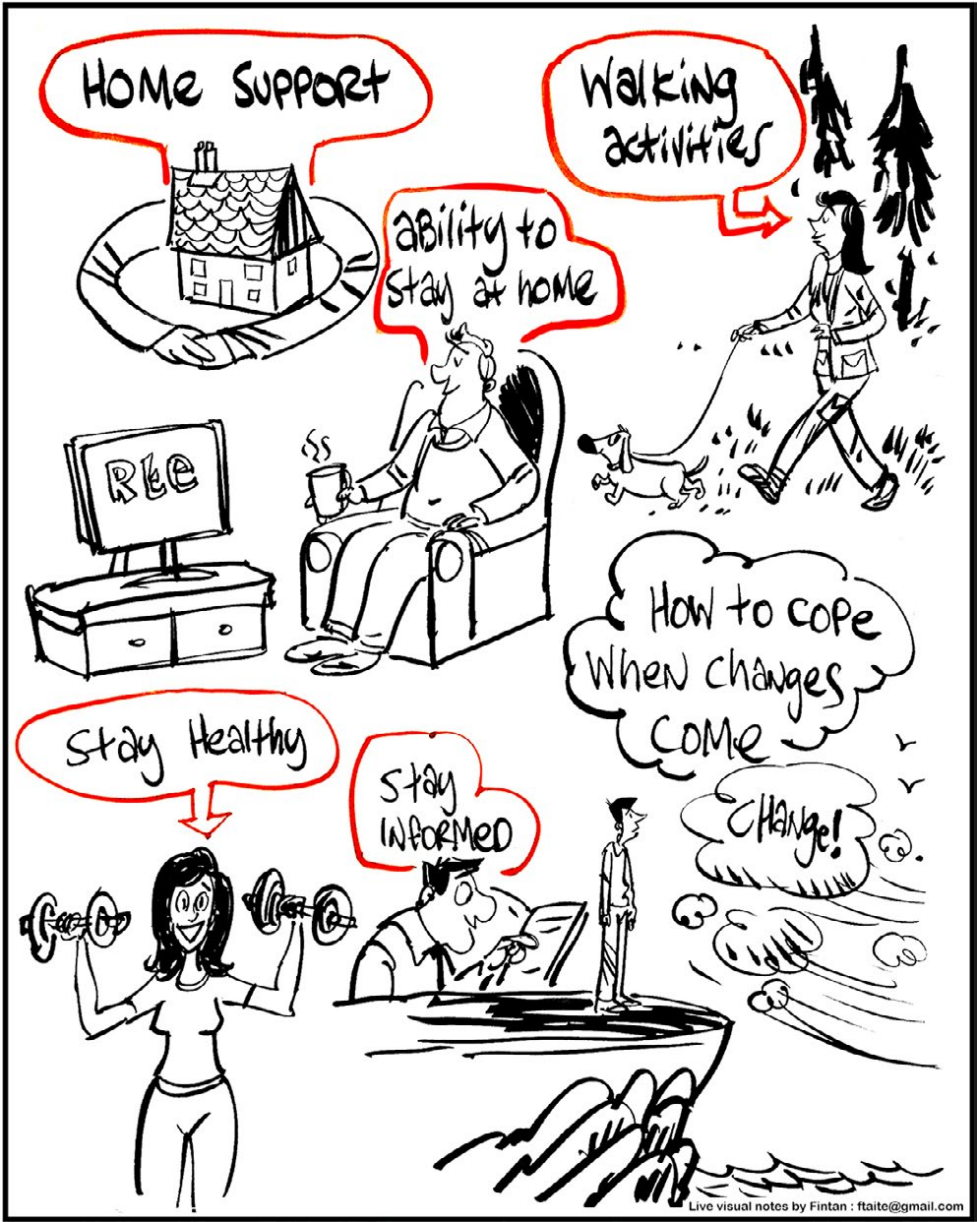
Example illustration from the policy café

Illustrations – As the illustrator worked, he posted his pictures on the walls of the meeting room. Participants checked these out during breaks and as they moved around to the other table. They reacted positively to the illustrations, recognizing the content of their discussions in the visual format. For example, in the illustration below, the artist incorporated an image which one of the participants had drawn in the course of the discussion to convey their view of home—with arms around the house (see Figure 2). The other illustrations are included as a supplementary file.

The filmmakers adopted a ‘fly‐on‐the‐wall’ style and did not intervene in the discussions or ask questions. They simply recorded what went on and participants got used to their presence very quickly. The filmmakers also conducted individual interviews with a number of participants after the event. Three films were made to convey the messages from the policy café; a film of the policy cafe and two films with three of the participants which contained supplementary material filmed in their homes.^31^

### 3.2 Carer's Assembly

The results of the ballot in each topic are shown in Table 4, presented in order as voted by the carers at the Assembly for each topic.

**TABLE 4:** Rank order of the top three attributes in the topics discussed at the Carer's Assembly

Rank	Desired attributes	Description
Home care		
1.	Dementia‐specific training (family and professional carers)	Dementia‐specific training should be provided to all involved in the care of a person with dementia. Well‐trained professional carers should have knowledge and understanding of dementia; and have skills to work with people with dementia in an effective and considerate way
2.	Access to quality home care	Access to statutory home care should be a right and the amount of care provided should be increased
3.	Continuity and quality of care (including regulation)	The same professional home carers should provide regular care to the person with dementia and home care should be provided consistently at scheduled times. Continuity of care should be provided country‐wide. There should be a clear set of standards that carer support agencies and workers have to adhere to
Respite services		
1.	In‐home respite	A trained professional should be available to supervise the person with dementia in their home to give family carers a short break during the day (not overnight)
2.	Day services	A day care service run by trained professionals for people with dementia to attend outside of the home, giving the family carer a short break during the day each week, as well as providing social engagement and activity for person with dementia
3.	Residential respite	Trained professionals would provide overnight care to the person with dementia for a period of 4 wk every year in a location outside of the home (eg a community hospital or care home) to provide family carers with a break from their caring responsibilities
Psychosocial supports		
1.	Information for carers	Information should be available to carers at the right time, in the right place, through the right medium. Easy access to information on carer supports should also be available
2.	Family carer education programme	A six session education course to develop family carers' knowledge of dementia and caring skills to enable carers to respond more confidently to the needs of the relative or friend you provide care to. This can be delivered online or in‐person
3.	Family carer support group	A monthly meeting which provides the opportunity to meet other family carers of people with dementia in a similar situation, to share experiences, and access information and practical advice in a relaxed environment
Financial supports		
1.	Non‐means tested Carer's Allowance	All family carers should receive a payment of up to €219 weekly for the care they provide to their relative with dementia. Currently the allowance system is means tested and covers approximately 20% of all carers
2.	Medical card for carers and people with dementia	A medical card providing free GP and hospital care and significantly subsidized prescriptions should be automatically provided to everybody diagnosed with dementia and their family carer
3.	Carer's Support Grant	An annual lump sum payment of €1,900 is provided to all qualifying carers and should continue to be provided under any new legislation

Finally, the four themes addressed on the day were ranked in order of priority and policy relevance by carers through a private ballot. The resultant priorities were identified in the following order:

1. homecare services
2. economic support
3. respite care
4. psychosocial supports and services.

Informal feedback was sought throughout the event. Overall, the feedback was positive; the participants felt heard and many reported, and for the first time as a family carer, they felt valued and had a voice. There was a clear focus on the day to make sure that all discussion was action and policy focused and having a professional, well‐experienced facilitator at each table enabled this to happen. The learning from the pilots ensured everything ran smoothly on the day.

The illustrator worked in a similar way to that described in the policy café and participants in the carer's assembly also reacted well to his illustrations. As with the policy café, the filmmakers adopted a ‘fly‐on‐the‐wall’ approach to recording the proceedings of the event and also conducted interviews with a number of carers immediately following the event.^31^

## 4 DISCUSSION

Policymaking is typically a top‐down bureaucratic process, with a concentration of power in government bodies and topic experts. In health policymaking, early moves to involve patient groups were often tokenistic and did not acknowledge the power imbalances and other challenges when, for example a single lay person became part of a policymaking group. The use of more inclusive methods such as citizen assemblies and citizen juries is not widespread.^32^ However, even these methods can be difficult for people with disabilities to access. The aim of this work was to move beyond tokenistic efforts of involvement and to develop bottom up, co‐produced methods for enabling the unfiltered voice of people with dementia and carers to be brought directly into the policymaking process.

Issues of power are at the heart of PPI and co‐production. Arnstein's ladder of involvement^10^ and frameworks which have followed^33^ generally describe a range of involvement from tokenism to full citizen control, illustrating greater or lesser levels of power or influence in decision making.^18^ The adoption of co‐production methods was designed to ensure power was shared between the participants (people with dementia and carers) and the researchers designing the events. The participants decided the topics were involved in the design of the events and identified their messages and priorities for policy‐makers in an unfiltered way. The use of co‐production throughout these processes affirmed the personhood of the participants and embedded the message of equality and shared power. This approach led to the use of non‐traditional recording and dissemination methods, such as film and illustration, to supplement the production of formal reports and papers. The use of film and illustration was embraced by participants as it provided a means to amplify their voice and was also an accessible account of the discussions, not just for people with dementia and carers but also for policy‐makers.

Inevitably, there are practical challenges in what can be achieved within these methods. The main challenge that emerged was how to balance the depth and breadth of topics and discussions within a reasonable time period. The participants with dementia identified a limit for a meeting in terms of concentration and effort of about 2‐2.5 hours. Given the effort in time and organization for the policy café, the participants wanted to cover more than one topic. While the Wold Café method worked well in many respects, the element where tables were swapped so that participants could review the themes identified by the other group did not work so well. An alternative would have been to omit this step altogether or to have a less in‐depth discussion of the two topics at each table. There is no ‘right’ answer here, although a more detailed ‘walk through’ of the event in advance may have helped to identify this issue.

A practical and valuable impact from the work has been the publication of guidelines on the involvement of people with dementia in policy, advisory, consultation and conference activities.^24^ Titled *Hear Our Voice*, these guidelines were co‐produced with people with dementia and have been presented widely at national and international conferences.^34^, ^35^

Generating impact on the policymaking process is an acknowledged challenge,^36^ particularly establishing and maintaining genuine engagement from policy‐makers.^37^ The policy café and carer's assembly were useful in themselves as stand‐alone events, generating policy relevant issues directly from those affected, and being valued by the participants who described personal benefits from their participation. However, in order to generate policy impact, these events were conceived and developed as part of a wider series of events, each building cumulatively on the other to generate maximum effect. A policy symposium on *Ageing, Social Care and Social Justice* ^38^ was the first event, engaging key stakeholders in an in‐depth exploration of critical questions on the future of social care for older people in Ireland. The policy café and the carer's assembly then took place some months after the symposium. The outputs (film, illustrations and documented priorities) from the café and the assembly were part of the materials for a policy dialogue on home care, co‐organized with the Department of Health.^39^, ^40^ A policy dialogue is a multi‐stakeholder event which bring research evidence together with the views, experiences and knowledge of those who will be involved in, or affected by, future decisions about high priority issues.^41^ While people with dementia, older people and carers participated in the policy dialogue, the use of the outputs from the café and assembly was a conscious effort to maximize the impact of these participants through a broader representation of the dementia experience. The new legislation for home care is still in the process of development, so it is too early to estimate the impact of these efforts on this specific policy. However, the commitment of senior policy‐makers from the health service, the Department of Health and other Government departments to the process has, in itself, been an important outcome of this work, specifically their willingness to engage with the concept of patient and public involvement in the decision‐making process.

The partnership approach embodied by co‐production developed over a period of time (more than 5 years) and extended beyond the participants directly involved. Thus, relationships were built over a period of years with key stakeholders such as senior officials in the health service and in the Department of Health, as well as with non‐governmental organizations such as the Alzheimer Society of Ireland, often through joint working on various initiatives. Real, long‐lasting change is achieved through working with the key actors in a respectful way, an approach that recognizes the agenda and realities of all participants and the futility of a zero sum approach to achieving shared goals. The two approaches reported in this paper, based around participation and dialogue, allow trust to build and change to follow. Partnership working and co‐production helps engage political power while working with the public servants, building a constituency for change and support for the longer term work of policy implementation.

## CONFLICT OF INTEREST

The authors have no conflict of interest to declare.

## Data Availability

All of the data generated in the course of the study are included in this article.
